# qPCR detection of Sturgeon chub (*Macrhybopsis gelida*) DNA in environmental samples

**DOI:** 10.1371/journal.pone.0209601

**Published:** 2018-12-31

**Authors:** Daniel H. Mason, Joseph C. Dysthe, Thomas W. Franklin, Joseph A. Skorupski, Michael K. Young, Kevin S. McKelvey, Michael K. Schwartz

**Affiliations:** 1 National Genomics Center for Wildlife and Fish Conservation, Rocky Mountain Research Station, United States Forest Service, Missoula, Montana, United States of America; 2 Wyoming Game and Fish Department, Cody, Wyoming, United States of America; National Zoological Park, UNITED STATES

## Abstract

The Sturgeon chub (*Macrhybopsis gelida*) is a cyprinid fish native to the Missouri and Mississippi River basins of the U.S. Suspected long-term declines in the size of its distribution have prompted a review of its conservation status by the U.S. Fish and Wildlife Service, a process which depends on reliable methods to delineate the distribution and status of extant populations. To facilitate monitoring of Sturgeon chub populations, we developed a quantitative PCR assay to detect Sturgeon chub DNA in environmental samples. The assay consistently detected Sturgeon chub DNA in concentrations as low as 2 copies per reaction, and did not amplify DNA from non-target fish species that are sympatric in the upper Missouri River basin. Field tests of this assay with environmental samples successfully detected Sturgeon chub from sites known to be occupied. This assay offers an extremely sensitive methodology that can be applied to determine the range of Sturgeon chub, regardless of variation in habitat characteristics.

## Introduction

The challenges of scientifically sampling for small-bodied or rare aquatic species are compounded in large aquatic systems, especially in riverine habitats (reviewed in [1]). Frequently, studies of organisms that potentially occupy both large and small stream habitats are forced to employ different sampling methods, with sampling efficiencies dictated by stream discharge and environmental conditions at different sampling sites [2], [3]. The use of disparate survey methods complicates the interpretation of survey results, which is problematic in cases where each observation of a species may be of substantial consequence for resource managers.

These circumstances describe the efforts to survey Sturgeon chub (*Macrhybopsis gelida*), a wide-ranging species which has been collected from large rivers (e.g. mean discharge >2,400 m^3^/s) and small tributaries (~11 m^3^/s) of 13 U.S. states within the Missouri and Mississippi River watersheds [4], including Montana (MT) and Wyoming (WY). Previous distributional and abundance surveys have relied on an array of sampling techniques, with predictably variable results. Several of these assessments have documented evidence of population declines compared to historical records [5], [3] but others have observed high relative abundances [6], [7], [8]. This ambiguity creates a challenge for science-based management and assessment of Sturgeon chub.

The U.S. Fish and Wildlife Service is currently assessing the Sturgeon chub as a candidate for listing under the Endangered Species Act (ESA; 16 U.S.C. § 1531 et seq.) [4]. Critically, much of the known distribution of the Sturgeon chub has been affected by the development and operation of large regulatory and hydroelectric dams, which alter the depth, flow, temperature, turbidity, substrate, water chemistry, and geomorphology of impounded and downstream reaches, as well as their fish communities [9], [10], [3], [4]. Riverine alterations have the potential to affect the persistence of Sturgeon chub across its entire range, while activities related to resource extraction may additionally threaten populations within the Missouri River system [4]. Thus, developing a rapid and reliable method for monitoring populations of Sturgeon chub across its range would be useful for evaluating the species’ status and prioritizing conservation efforts.

Environmental DNA (eDNA) sampling is an efficient and reliable method for delineating distributions of rare species [11], detecting taxa that are sensitive to disturbance [12], [13], [14] or surveying for species that are difficult to detect by direct observation [15]. Furthermore, eDNA methods are strengthened by employing quantitative PCR (qPCR), which is more sensitive and effective at detecting low DNA concentrations than end-point PCR [16], [17]. Accordingly, we developed a qPCR assay for eDNA-based detection of Sturgeon chub in the Upper Missouri River basin in WY and MT.

## Methods

To develop an eDNA assay for detecting Sturgeon chub, we examined a partial sequence of the cytochrome *b* (*cytb*) mitochondrial gene region available from GenBank, as well as *cytb* data from 14 non-target species that are closely related or sympatric (Table 1). Due to the lack of previously published genetic data, we generated ten additional *cytb* sequences from Sturgeon chub collected from the Missouri River in MT (*n* = 2) and Powder River in WY (*n* = 8) (Table 1). To bolster sampling of non-target taxa, we also generated *cytb* sequences from Sicklefin chub (*Macrhybopsis meeki*, *n* = 2) from the Missouri River in MT and Flathead chub (*Platygobio gracilis*, *n* = 5) collected from the Missouri River in MT and the Powder River in WY (Table 1). A small fin clip of approximately 1 cm in length and width was taken from the caudal fin of each fish before they were quickly released. Fin clips were collected under the auspices of the Wyoming Game and Fish Regulation; Chapter 56, thus additional permits or ethical review were not required. Fin clips were stored in ≥95% ethanol until DNA was extracted using the DNeasy Blood & Tissue Kit (Qiagen, Inc). Prior to extraction, we bleached the tissues with a 10% sodium hypochlorite solution to remove eDNA from co-occurring species that may have been on the tissue surface, then thoroughly rinsed each tissue with DI water to minimize destruction of target DNA. PCR products for sequencing were amplified using forward primer: 5’–CCTATGACTTGAAGAAACATCGTTG– 3’ and reverse primer: 5’–CCCTCAATCTTCGGATTACAAGAC– 3’. Primers were modified from the primers L14724 and H15915 as designed in [18] by aligning them in MEGA 7 [19] with a nearly complete mitogenome of Sturgeon chub (accession: AP012080.1) and manually adjusting nucleotides to identically match the Sturgeon chub sequence. PCR products were generated in 40 μl reactions consisting of 4μl (~4–20 ng) DNA template, 4 μl of 10X PCR buffer, 4 μl MgCl_2_ (2.5 mM), 1 μM of each primer, 200 μM each dNTP, 25 μg BSA, 1 Unit Titanium Taq DNA Polymerase (Takara Bio USA, Inc), and the remainder with PCR grade distilled water. The thermocycling conditions contained an initial denaturation at 95 °C for 12 min, followed by 35 cycles of denaturation at 94 °C for 1 min, annealing at 55 °C for 1 min, and extension at 72 °C for 1.5 min; there was a final extension stage at 72 °C for 5 min. PCR products were cleaned using ExoSAP-IT PCR Product Cleanup Reagent (Life Technologies) and sequences were generated on an ABI 3730XL sequencing machine at Eurofins Genomics. We processed the raw sequencing data in Sequencher v 5.4.6 (Gene Codes Corporation) and trimmed them to the 1140 base *cytb* gene.

**Table 1:** *In silico* assay validation.

	Species name	Common name	*n*	GenBank accession	Mismatches		
					Forward primer	Reverse primer	Probe
Targets	*Macrhybopsis gelida*	Sturgeon chub	1	AP012080.1	0	0	0
			10*	MK291379-88	0	0	0
Non-targets	*Couesius plumbeus*	Lake chub	1	AY281053.1	4	4	5
	*Ctenopharyngodon idella*	Grass carp	1	AF420424.1	2	5	6
	*Cyprinella lutrensis*	Red shiner	1	GQ275188.1	4	3	6
	*Cyprinus carpio*	Common carp	1	FJ478021.2	1	6	4
	*Hybognathus argyritis*	Western silvery minnow	1	EU811094.1	2	2	2
	*Hybognathus hankinsoni*	Brassy minnow	1	AF452080.1	2	3	5
	*Hybognathus placitus*	Plains minnow	1	EU082470.1	5	3	3
	*Luxilus cornutus*	Common shiner	1	U66597.1	4	2	2
	*Machrybopsis aestivalis*	Spotted chub	1	JQ712319.1	4	3	5
	*Machrybopsis meeki*	Sicklefin chub	1	NC_033936.1	1	4	2
			2*	MK291389-90	1	4	2
	*Margariscus margarita*	Pearl dace	1	AF452072.1	2	6	7
	*Nocomis biguttatus*	Hornyhead chub	1	AY486057.1	3	2	5
	*Notemigonus crysoleucas*	Golden shiner	1	NC_008646.1	4	6	6
	*Notropis atherinoides*	Emerald shiner	1	AY096008.1	3	3	2
	*Notropis hudsonius*	Spottail shiner	1	KT834523.1	4	5	4
	*Notropis stramineus*	Sand shiner	1	HM179637.1	2	4	4
	*Phoxinus neogaeus*	Finescale dace	1	EU755059.1	6	4	3
	*Pimphales promelas*	Fathead minnow	1	GQ184519.1	4	4	2
	*Platygobio gracilis*	Flathead chub	1	JX442992.1	1	2	3
			5*	MK291391-95	1	2	3
	*Rhinichthys cataractae*	Longnose dace	1	DQ990251.1	2	4	8
	*Semotilus atromaculatus*	Creek chub	1	AF452082.1	4	6	4

Species, sample size (*n*), and GenBank accession number for DNA sequences used for *in silico* Sturgeon chub assay development.

*An asterisk denotes sequences generated at the National Genomics Center for Wildlife and Fish Conservation (NGCWFC), USDA Rocky Mountain Research Station, Missoula, Montana

We aligned all sequences in MEGA 7 [19] and used Primer-BLAST [20] to identify candidate primer sites that would amplify a 102-nucleotide region in our alignment that was unique to sturgeon chub (Table 2). Within this fragment, we designed a FAM-labeled, minor-groove-binding, non-fluorescent quencher (MGB-NFQ) probe (Table 2). We maximized within-primer and within-probe nucleotide mismatches with respect to non-target sequences to avoid instances of primer competition and cross-amplification of the probe [16]. We used Primer Express 3.0.1 (Life Technologies) to adjust primer and probe lengths to optimize annealing temperatures and screened them for secondary structures using the IDT OligoAnalyzer web application (https://www.idtdna.com/calc/analyzer). Using the NCBI nucleotide BLAST tool, we further examined the specificity of the assay *in silico* to reduce the potential for detecting non-target taxa. Each oligonucleotide was examined individually in this manner before the complete assay was assessed using Primer BLAST and the full NCBI nucleotide collection.

**Table 2:** Sturgeon chub eDNA assay.

Assay component	Sequence (5’-3’)	Tm (°C)	Optimal concentration (nM)
Forward primer	CTAACATGAATTGGAGGCATACCA	58.9	300
Reverse primer	CGAGTGGGGCAAGGATGA	59.1	300
Probe	FAM-CCTCAGTTCTATACTTTGCACTAT-MGBNFQ	70	250

Primers and hydrolysis probe for detecting Sturgeon chub using qPCR.

We tested the specificity of the assay *in vitro* using a QuantStudio 3 Real-time PCR Instrument (Life Technologies) in 15-μl reactions containing 7.5 μl Environmental Master Mix 2.0 (Life Technologies), 300 nM each forward and reverse primer, 250 nM of probe, 4 μl DNA template (~0.4 ng), and PCR-grade water for the remaining volume. Thermocycler conditions were 95 °C for 10 min followed by 45 cycles of denaturation at 95 °C for 15 s and annealing at 60 °C for 1 min. Pipettes, tube racks, and consumables were irradiated with UV light in a hood for 1 h prior to set-up. We screened DNA extracted from 25 Sturgeon chub tissues collected at two locations, and from 40 additional non-target species (Table 3). DNA used for *in vitro* screening was obtained from archival samples, or from small fin clips. Fin clips were extracted following the same methods as described above for sequencing. DNA extracts were quantified with a Qubit 2.0 fluorometer and diluted to 0.1 ng/μl of genomic DNA before testing.

**Table 3:** *In vitro* assay validation.

Family	Species name	Common name	Sample size	Origin
Cyprinidae	*Macrhybopsis gelida*	Sturgeon chub	2	Missouri River, MT
			23	Powder River, WY
Acipenseridae	*Scaphirhynchus platorynchus*	Shovelnose sturgeon	1	Yellowstone River, MT
Catostomidae	*Carpiodes carpio*	River carpsucker	1	Missouri River, MT
	*Catostomus catostomus*	Longnose sucker	1	Missouri River, MT
	*Catostomus commersonii*	White sucker	2	MT; Rio Grande, NM
	*Catostomus jordani*	Mountain sucker	1	MT
	*Catostomus platyrhynchus*	Mountain sucker	1	Gros Ventre River, WY
	*Ictiobus bubalus*	Smallmouth buffalo	1	Yellowstone River, MT
	*Moxostoma macrolepidotum*	Shorthead redhorse	1	Missouri River, MT
Centrarchidae	*Micropterus dolomieu*	Smallmouth bass	2	Clark Fork River, MT
	*Micropterus salmoides*	Largemouth bass	1	MT
	*Pomoxis annularis*	White crappie	1	Missouri River, MT
	*Pomoxis nigromaculatus*	Black crappie	1	MT
Cyprinidae	*Cyprinella lutrensis*	Red shiner	1	Gila River, NM
	*Cyprinus carpio*	Common carp	1	North Platte River, WY
	*Hybognathus argyritis*	Western silvery minnow	1	MT
	*Macrhybopsis meeki*	Sicklefin chub	3	Missouri River, MT
	*Notropis atherinoides*	Emerald shiner	1	Missouri River, MT
	*Notropis stramineus*	Sand shiner	1	Missouri River, MT
	*Pimephales promelas*	Fathead minnow	2	Missouri River, MT; Gila River, NM
	*Platygobio gracilis*	Flathead chub	7	Missouri River, MT; Powder River, WY
	*Rhinichthys cataractae*	Longnose dace	2	Tin Cup Creek, ID; Missouri River, MT
	*Semotilus atromaculatus*	Creek chub	1	NM
Esocidae	*Esox lucius*	Northern pike	2	Missouri River, MT
Hiodontidae	*Hiodon alosoides*	Goldeye	1	Missouri River, MT
Ictaluridae	*Ictalurus natalis*	Yellow bullhead	1	MT
	*Ictalurus punctatus*	Channel catfish	1	MT
	*Noturus flavus*	Stonecat	1	Missouri River, MT
Lotidae	*Lota lota*	Burbot	1	MT
Percidae	*Perca flavescens*	Yellow perch	1	Yellowstone River, MT
	*Sander canadensis*	Sauger	1	Missouri River, MT
	*Sander vitreus*	Walleye	1	Bighorn Lake, WY
Salmonidae	*Coregonus clupeaformis*	Lake whitefish	1	Saint Mary Lake, MT
	*Oncorhynchus clarki bouvieri*	Yellowstone cutthroat trout	1	Yellowstone Lake, WY
	*Oncorhynchus clarkii lewisi*	Westslope cutthroat trout	1	Rock Creek, MT
	*Oncorhynchus mykiss gairdneri*	Redband rainbow trout	1	Ruby Creek, MT
	*Salmo trutta*	Brown trout	1	South Little Tongue River, WY
	*Salvelinus fontinalis*	Brook trout	1	East Fork Weiser River, ID
	*Salvelinus namaycush*	Lake trout	1	Saint Mary Lake, MT
Sciaenidae	*Aplodinotus grunniens*	Freshwater drum	1	Yellowstone River, MT
	*Umbra limi*	Central mudminnow	1	Beaver Creek, MT

Species and sample sizes used for *in vitro* testing of the Sturgeon chub assay. Origin refers to the waterbody and U.S. state for all samples, except where waterbody information is not available.

We optimized primer concentrations by testing a single Sturgeon chub DNA sample with concentrations of each primer at 100, 300, 600, and 900 nM for a total of 16 unique assay concentrations [21]. We selected the assay concentration that displayed a high relative end-point fluorescence and the lowest C_t_ value for use in subsequent analyses. Using the optimal concentrations of 300 nM of both forward and reverse primer and the same qPCR conditions as above, we tested assay sensitivity and efficiency by analyzing a seven-level standard curve created from target qPCR product that was purified using a GeneJET PCR Purification Kit (Life Technologies), and quantified on a Qubit 2.0 fluorometer. We then converted the concentration from the fluorometer to DNA copy number by estimating the molecular weight of 1 mol of the double stranded, linear amplicon via the Sequence Manipulation Suite web application (http://www.bioinformatics.org/sms2/dna_mw.html). We used Avogadro’s number to estimate the copies per μl of the concentrated qPCR product and serially diluted it in sterile TE to 31 250, 6 250, 1 250, 250, 50, 10, and 2 copies per 4 μl. This standard curve was analyzed across six replicates of each level on a single 96-well qPCR plate.

Finally, we validated the assay *in vivo* by screening eDNA samples collected from two streams in the western U.S. with known patterns of occupancy by Sturgeon chub (Table 4). The eDNA samples were collected by filtering 5 L of water using methods outlined in [22]. DNA was extracted from the filters with the DNeasy Blood & Tissue Kit (Qiagen, Inc) following a protocol optimized for stream eDNA samples [23], including an extraction negative. Using the optimized qPCR conditions, the extracts were then analyzed along with a TaqMan Exogenous Internal Positive Control (1.5 μl of 10X IPC assay and 0.15 μl of 50X IPC DNA per reaction; Life Technologies), to screen for qPCR inhibition by environmental contaminants. All eDNA samples were analyzed *in vivo* in triplicate along with no-template qPCR negative controls.

**Table 4:** *In vivo* assay development.

Waterbody	Site #	UTM Zone	Easting	Northing	Collection date	Sturgeon chub expected	Sturgeon chub DNA detected	DNA copies per liter
Clear Creek	01	13T	411477	4934493	8/7/2017	n	n	0
	02	13T	411425	4964493	8/7/2017	n	n	0
	03	13T	410951	4964417	8/7/2017	n	n	0
	04	13T	410949	4964417	8/7/2017	n	n	0
	05	13T	410152	4962474	8/7/2017	n	n	0
	06	13T	410146	4962473	8/7/2017	n	n	0
	07	13T	409790	4962623	8/7/2017	n	n	0
	08	13T	409789	4962617	8/7/2017	n	n	0
	09	13T	409472	4962392	8/7/2017	n	n	0
	10	13T	409468	4962390	8/7/2017	n	n	0
Powder River	01A	13T	427380	4979685	8/7/2017	y	y	7.10
	01B	13T	427380	4979685	8/7/2017	y	y	9.62
	03A	13T	427235	4979619	8/7/2017	y	y	11.73
	03B	13T	427235	4979619	8/7/2017	y	y	16.33
	05A	13T	427077	4979548	8/7/2017	y	y	26.85
	05B	13T	427077	4979548	8/7/2017	y	y	42.92
	07A	13T	424944	4976018	8/7/2017	y	y	31.37
	07B	13T	424944	4976018	8/7/2017	y	y	28.06
	09A	13T	411897	4928531	8/8/2017	y	y	42.90
	09B	13T	411897	4928531	8/8/2017	y	y	68.77
	11A	13T	407619	4896780	8/9/2017	y	y	53.35
	11B	13T	407619	4896780	8/9/2017	y	y	29.70
Holding tank*	13A				8/10/2017	y	y	97224.19

Collection information and detection results for *in vivo* testing of the Sturgeon chub assay. All samples were collected in Wyoming.

*This sample was collected from a holding tank

## Results & discussion

**Fig 1:**
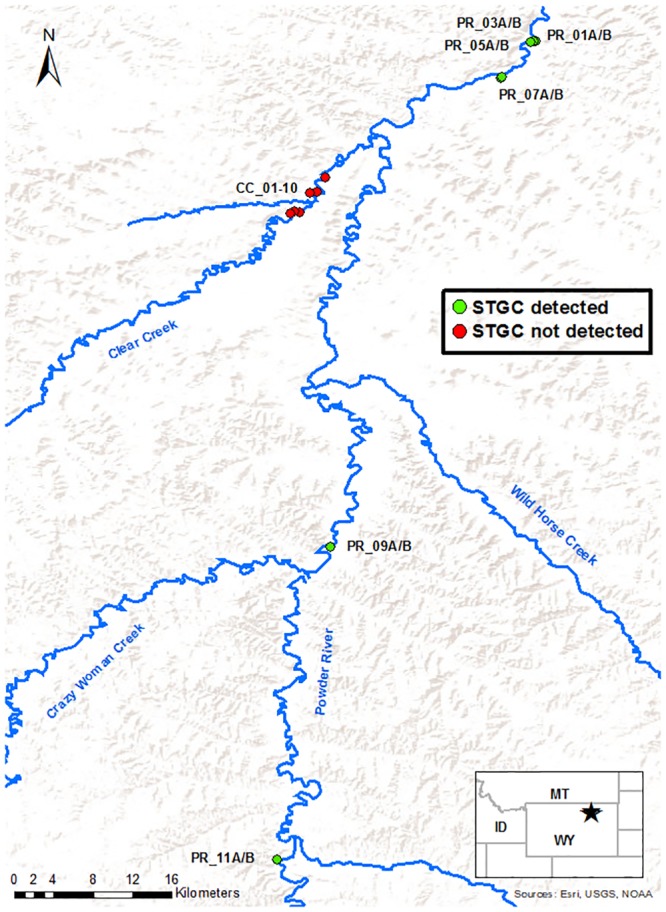
Map of Sturgeon chub (STGC) eDNA sampling sites. Sturgeon chub were not detected in 10/10 samples from reaches where they were known to be absent, and were detected in 12/12 samples from reaches where they were known to be present. An additional positive sample collected in the laboratory from a Sturgeon chub holding tank is not shown.

The assay detected DNA from all Sturgeon chub tissue samples and did not detect DNA from the non-target species or within the no-template controls. The standard curve demonstrated a reaction efficiency of 95.572% (*R*^*2*^ = 0.991, y-intercept = 37.549, slope = -3.433) and a limit of detection (defined here as the lowest concentration with > 95% amplification success; [24]) that was equal to or less than 2 copies per reaction; DNA was detected in all six replicates at this concentration. Finally, Sturgeon chub DNA was not detected in any environmental samples taken where the species was expected to be absent, and was detected in all samples where the species was expected or known to be present (Table 4, Fig 1). We did not, however, evaluate this assay for application in the Mississippi River basin, nor did we test its specificity across taxa with which it could co-occur in that region. Further assay evaluation would be necessary before it could be applied in geographically distant drainages or in areas with different aquatic assemblages.

This qPCR assay reliably detected low concentrations of Sturgeon chub DNA and did not detect the DNA of non-target fish species known from the upper Missouri River basin. As such, eDNA sampling for Sturgeon chub should be extremely sensitive, as long as robust protocols [22], [11] are paired with field surveys that address the ecological characteristics influencing the distribution of this species [2], [25], [26]. For instance, Sturgeon chub tend to occupy turbid stream environments, which can be difficult to sample using traditional methods. However, eDNA surveys for sturgeon chub in the Powder River in WY, a stream with very high turbidity, were highly effective. Results from such surveys could help biologists target their conservation efforts and more effectively evaluate the success of management activities.
